# Pentapeptide-Zinc Chelate from Sweet Almond Expeller Amandin Hydrolysates: Structural and Physicochemical Characteristics, Stability and Zinc Transport Ability In Vitro

**DOI:** 10.3390/molecules27227936

**Published:** 2022-11-16

**Authors:** Jiangning Zhang, Zheng Ye

**Affiliations:** Shanxi Functional Food Research Institute, Shanxi Agricultural University, Taiyuan 030810, China

**Keywords:** sweet almond expeller amandin, peptide-zinc chelate, physicochemical characterisation in silico, security prediction, stability, transport

## Abstract

To promote the application of almond expellers, sweet almond expeller globulin (amandin) was extracted for the preparation of bioactive peptides. After dual enzymatic hydrolysis, Sephadex G-15 gel isolation, reverse-phase high-performance liquid chromatography purification and ESI-MS/MS analysis, two novel peptides Val-Asp-Leu-Val-Ala-Glu-Val-Pro-Arg-Gly-Leu (1164.45 Da) and Leu-Asp-Arg-Leu-Glu (644.77 Da) were identified in sweet almond expeller amandin hydrolysates. Leu-Asp-Arg-Leu-Glu (LDRLE) of excellent zinc-chelating capacity (24.73 mg/g) was selected for preparation of peptide-zinc chelate. Structural analysis revealed that zinc ions were mainly bonded to amino group and carboxyl group of LDRLE. Potential toxicity and some physicochemical properties of LDRLE and Val-Asp-Leu-Val-Ala-Glu-Val-Pro-Arg-Gly-Leu (VDLVAEVPRGL) were predicted in silico. The results demonstrated that both LDRLE and VDLVAEVPRGL were not toxic. Additionally, zinc solubility of LDRLE-zinc chelate was much higher than that of zinc sulphate and zinc gluconate at pH 6.0–10.0 and against gastrointestinal digestion at 37 °C (*p* < 0.05). However, incubation at 100 °C for 20–60 min significantly reduced zinc-solubility of LDRLE-zinc chelate. Moreover, the chelate showed higher zinc transport ability in vitro than zinc sulphate and zinc gluconate (*p* < 0.05). Therefore, peptides isolated from sweet almond expeller amandin have potential applications as ingredient of zinc supplements.

## 1. Introduction

About one third of the global population, especially pregnant women and children, is zinc deficient [1]. Zinc deficiency can cause anorexia, an immunocompromised state, growth retardation and cognitive impairment for people, especially infants and children [2]. In recent years, food protein-derived peptide-zinc chelate has received attentions because it is more easily absorbed and more stable against gastrointestinal digestion and interference by other nutrients. Moreover, peptide-zinc chelate has no side effect in comparison with other inorganic or organic zinc supplements [3]. Furthermore, some peptide-zinc chelates showed healthcare benefits for humans, such as a hypolipidemic effect, resistance to radiation and hypoglycemic effect [4]. It is thought that, regarding bioavailability in vivo, the zinc fortification effect of peptide-zinc chelate is related to adsorption mechanisms, security, physicochemical properties, and stability under different conditions and against the gastrointestinal barrier [5,6]. Some gastrointestinal proteases can degrade the amino acid sequence of bioactive peptides and cause collapse of peptide-zinc chelate structure [7]. Moreover, some food-processing technologies such as thermal treatment, acid or alkali treatment, and reaction with sugar, salt or other nutrients can reduce the zinc solubility and absorption rate of peptides-zinc chelate [8]. Additionally, potential allergenicity or toxicity means that peptides-zinc chelate is not fit for use in the food or pharmaceutical industries [9]. Apart from these, physicochemical properties, especially hydrophilicity and hydrophobicity, significantly affect the zinc-chelating ability of peptides and applications of peptide-zinc chelate in various food systems [10]. Although an increasing number of studies have focused on preparation, absorption mechanisms and in vivo bioavailability of food-derived peptide-zinc chelates, few data have referred to the physicochemical properties, security and stability of peptide-zinc chelate.

Sweet almond (*Prunus Amygdalus dulcis*) expeller is the main byproduct of sweet almond oil manufacture and contains high levels of protein (around 37 g/100 g) [11]. The annual production of sweet almond is about 1.7 million tons worldwide [12]. Moreover, the yield of sweet almond expeller has increased with increasing worldwide almond production and demand for apricot oil. Almond protein has a relatively desirable amino acid composition and good emulsifying, foam and gelling properties [13,14,15]. In addition, some bioactive peptides such as antioxidant peptide, hypoglycemic peptide and hypotensive peptide have been identified in almond protein hydrolysates [16,17,18]. Previous study revealed that globulin (amandin) was the predominant fraction in almond protein (accounting for 65–70 g/100 g). However, bitter almond amandin is of allergenic potential [19]. The pre-experiment of this study found that sweet almond amandin possessed good zinc-chelating ability (16.32 mg/g). The main reason is that sweet almond amandin is a typical hexamer (360 kDa) consisting of six subunits which are linked by disulfide bonds [20]. Moreover, amandin is rich in acidic amino acids (Glu and Asp, 27.37 and 9.28 g/100 g, respectively) and sulfur amino acids (including Met and Cys, 2.67 g/100 g) [18]. The disulfide bond, acidic amino acids (Asp and Glu, both contain γ-carboxyl group) and sulfur amino acids (containing sulfhydryl) are all ideal chelating sites for metal ions [21]. Therefore, the objectives of the current study are to (i) identify peptides of excellent zinc-chelating capacity from sweet almond expeller amandin hydrolysates (SAEAH) and in silico prediction of security and physicochemical properties of the identified peptides; (ii) study the optimum preparation conditions, structure characteristics and zinc transportation capacity of sweet almond expeller peptides-zinc chelate; and (iii) investigate the stability of peptides-zinc chelate against different food-processing conditions and gastrointestinal digestion.

## 2. Results and Discussion

### 2.1. Isolation and Purification of Peptides of Excellent Zinc-Chelating Ability

**Figure 1:**
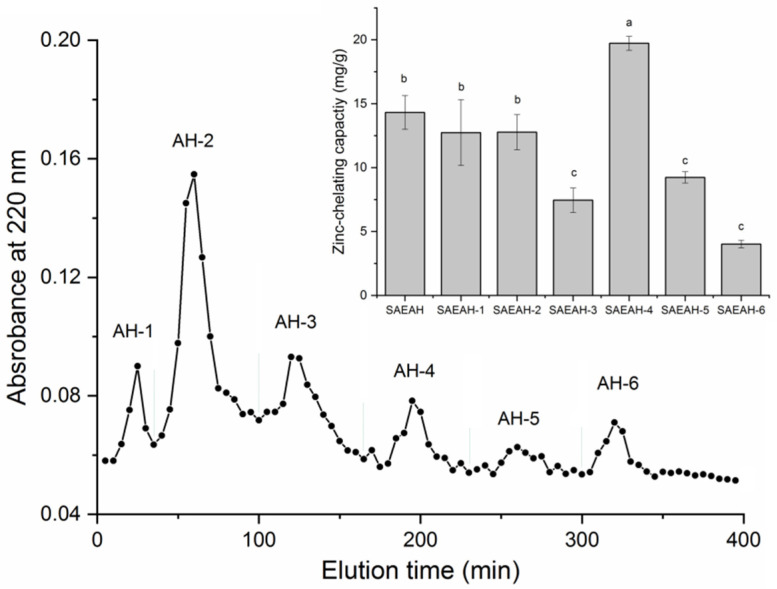
The separation profiles of sweet almond expeller amandin hydrolysates (SAEAH) on Sephadex G-15 gel chromatography and zinc-chelating capacities of subfractions AH-1, AH-2, AH-3, AH-4, AH-5 and AH-6. Lowercase letters (**a**–**c**) above the bars and in the table denote significant difference (*p* < 0.05).

**Figure 2:**
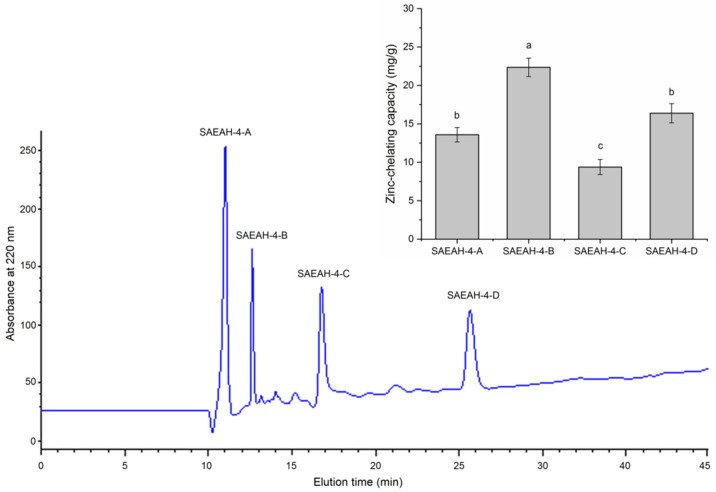
RP-HPLC profiles of fraction SAEAH-4 and zinc-chelating capacities of subfractions SAEAH-4-A, SAEAH-4-B, SAEAH-4-C and SAEAH-4-D. Lowercase letters (**a**–**c**) above the bars and in the table denote significant difference (*p* < 0.05).

After digestion with dual enzymes (Flavourzyme:Alcalase = 1:2), the hydrolysis degree of sweet almond expeller amandin was 32.67% ± 4.56%, which was consistent with the result of Li et al. [22]. The zinc-chelating ability of SAEAH was 14.32 ± 1.32 mg/g (Figure 1). After ultrafiltration with a membrane of 0.45 μm and Sephadex G-15 gel purification, six major subfractions were isolated from SAEAH. SAEAH-4 showed the highest zinc-chelating ability among these subfractions, so it was pooled, freeze-dried and analysed using reverse-phase high-performance liquid chromatography. The RP-HPLC profile of SAEAH-4 is shown in Figure 2. Obviously, there were four major subfractions isolated from SAEAH-4. Since SAEAH-4-B exhibited better ability in zinc-chelating than SAEAH-4-A, SAEAH-4-C and SAEAH-4-D (*p* < 0.05), SAEAH-4-B was pooled, freeze-dried and used for analysis of peptide sequences.

### 2.2. Characteristics of Peptide Sequence

**Figure 3:**
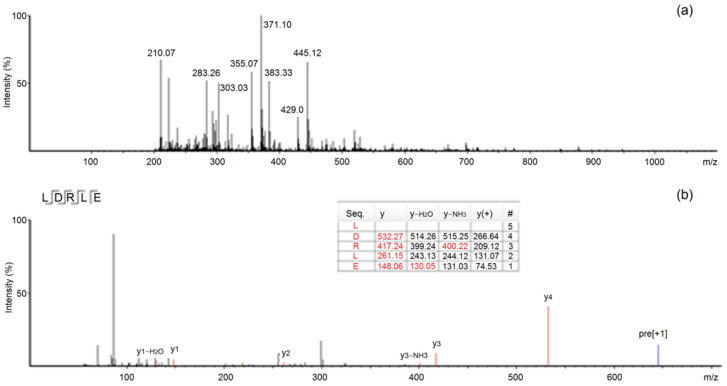
The primary (**a**) and secondary ESI-MS/MS spectra (**b**) of peptide LDRLE identified in sweet almond expeller amandin hydrolysates.

According to the mass spectrometry data, peptides Leu-Asp-Arg-Leu-Glu and Val-Asp-Leu-Val-Ale-Glu-Val-Pro-Arg-Gly-Leu were identified in SAEAH-4-B. The molecular weights of these peptides are shown in Table 1. The primary and secondary ESI-MS/MS spectra of peptide LDRLE are shown in Figure 3. LDRLE showed excellent zinc-chelating ability (24.73 mg/g); while VDLVAEVPRGL exhibited very poor zinc-chelating ability (4.33 mg/g). Therefore, LDRLE was selected for preparation of peptide-zinc chelate. Studies on structure-chelating ability relationship of oligopeptides revealed that zinc ions could effectively bond to groups of relatively strong negative polarity in peptides such as γ-carboxyl group (–COOH) and ε-amino group (–NH_2_) [21]. Moreover, free sulfhydryl of Cys residue and guanidino group of Arg residue in peptides were also the main chelating sites for zinc ions [23]. In the case of LDRLE, the Asp, Glu and Arg residues were mainly responsible for its excellent zinc-chelating capacity. Although VDLVAEVPRGL contained Asp and Glu residues, the larger molecule weight (1164.45 Da) reduced its zinc-chelating ability. Previous studies found that peptide of short chain always showed higher zinc-chelating ability than peptides of larger molecular weight [2,24]. Moreover, large peptides cannot pass through the gastrointestinal barrier to exert activity in vivo [10].

### 2.3. Physicochemical Characteristics and Toxicity Analysis of SAEAH Peptides

As shown in Table 1, the hydrophilicity of LDRLE (1.08) was much higher than that of VDLVAEVPRGL (*p* < 0.05), mainly attributed to its high content of hydrophilic amino acids (60%, Table 1). Moreover, the high hydrophilicity was responsible for the excellent zinc-chelating capacity of LDRLE (24.73 mg/g, Table 1). This is because polar amino acids such as Glu, Asp and Arg in peptides could chelate with zinc ions through ionic bonds or negative charge attraction [25]. The isoelectric point (pI) of LDRLE was 4.38, mainly attributed to the high content of Asp (pI of 2.97) and Glu (pI of 3.22). At the isoelectric point, the net charge on protein surface was zero and peptides tend to clump together, thereby reducing the electrostatic attraction between peptides and zinc ions, resulting in decrease in adsorption of zinc ions on peptides [10]. Moreover, the high amphiphilicity of LDRLE (0.74) meant that it had potential applications in emulsion food systems [7]. In addition, the in silico predicted result demonstrated that LDRLE and VDLVAEVPRGL were not toxic peptides. However, more studies in vitro and in vivo regarding the safety of these peptides are needed.

### 2.4. Structural Characteristics

#### 2.4.1. Scanning Analysis with Ultraviolet Wavelength

**Figure 4:**
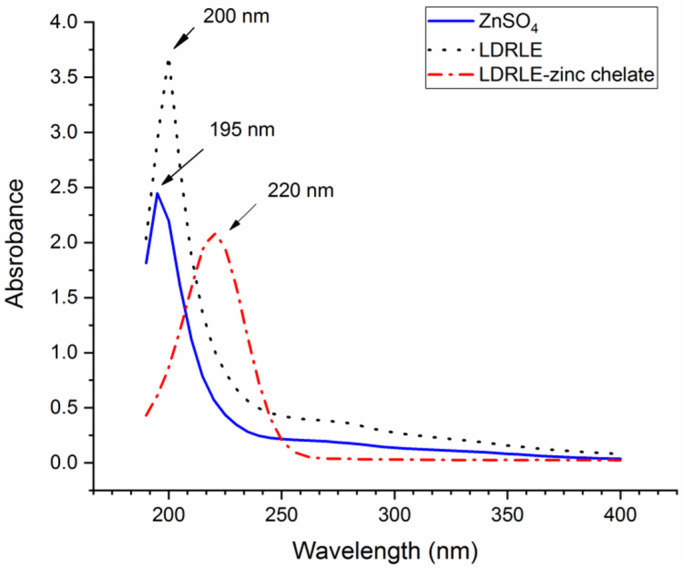
Ultraviolet spectra of LDRLE-zinc chelate and LDRLE.

As shown in Figure 4, the ultraviolet adsorption peaks of LDRLE and ZnSO_4_ were at 200 and 195 nm, respectively. The ultraviolet adsorption peak of LDRLE moved from 200 nm to 220 nm after the zinc chelation. This redshift on the adsorption peak demonstrated a combination between zinc ions and LDRLE [2]. Chelation with metal ions such as zinc or ferrous ions can cause electronic transitions in peptide molecules or changes in some chromophoric groups, thereby resulting in red or blue shifts of the ultraviolet adsorption peak of peptides [25]. A similar trend was noted by Sun et al. [6].

#### 2.4.2. FT-IR Analysis

**Figure 5:**
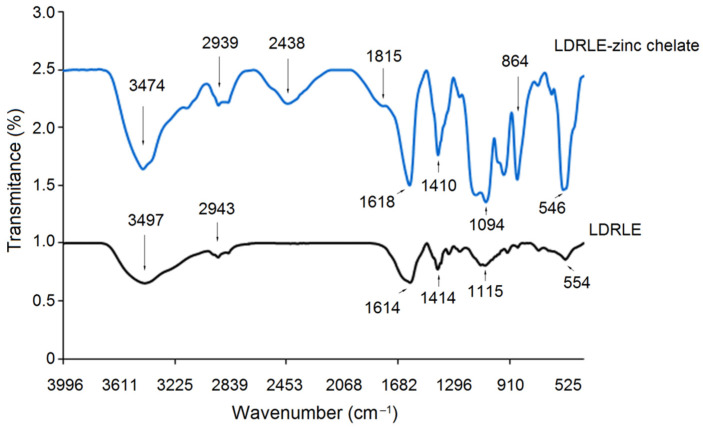
Fourier-transformed infrared spectra of LDRLE-zinc chelate and LDRLE.

FT-IR spectra of pure LDRLE and LDRLE-zinc chelate are shown in Figure 5. Significant differences were found on FT-IR spectrogram of LDRLE-zinc chelate in comparison with that of pure LDRLE. After zinc chelation, the adsorption peak of LDRLE occurred at 3497 cm^−1^ (indicative of the stretching of –N–H) moved to 3474 cm^−1^ [26]. In addition, a new adsorption peak appeared at 2438 cm^−1^ (reflecting the vibration of –C–N– bond) after the zinc chelation. These results suggested that zinc ions have been chelated with amide bond of LDRLE [4]. Moreover, adsorption peaks at 2943 cm^−1^ and 1614 cm^−1^ in the spectrum of LDRLE (both corresponding to the stretching of –C = O) shifted to 2939 and 1618 cm^−1^, respectively. In addition, new peaks appeared at 1815 and 864 cm^−1^ (representing the deformation vibrations of –C–O– and –COO–, respectively) after zinc chelation, demonstrating that the zinc ions have bonded to the carboxyl group of LDRLE [26]. In general, zinc ions mainly bonded to the amino group and carboxyl group of LDRLE.

#### 2.4.3. Microstructure

**Figure 6:**
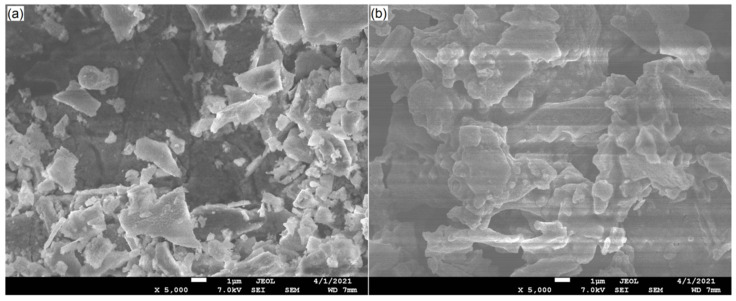
Scanning electron micrographs of LDRLE (**a**) and LDRLE-zinc chelate (**b**) with magnification of 5000× and a scale bar of 1 μm.

The microstructure of LDRLE was irregular and rougher and numerous loose fragments accumulated on the surface (Figure 6a). In comparison, the microstructure of LDRLE-zinc chelate was more compact with aggregated particles on the surface (Figure 6b), suggesting that the zinc chelation promoted the aggregation of LDRLE. It has been demonstrated that intermolecular aggregation may occur between some active groups of proteins in aqueous solutions. These active groups, such as the carboxyl group, sulfhydryl group and amino group, can bond with each other through hydrogen bonds [27]. A previous study found that zinc chelation can promote this aggregation [3]. After zinc chelation, the hydrophilic groups of peptides participating in the chelation with zinc ions will be hidden inside the peptide chains. This trend was beneficial for the intermolecular aggregation of peptides [27].

### 2.5. Stability Profiles of LDRLE-Zinc Chelate

#### 2.5.1. Effects of Thermal Treatment on Zinc-Solubility

**Figure 7:**
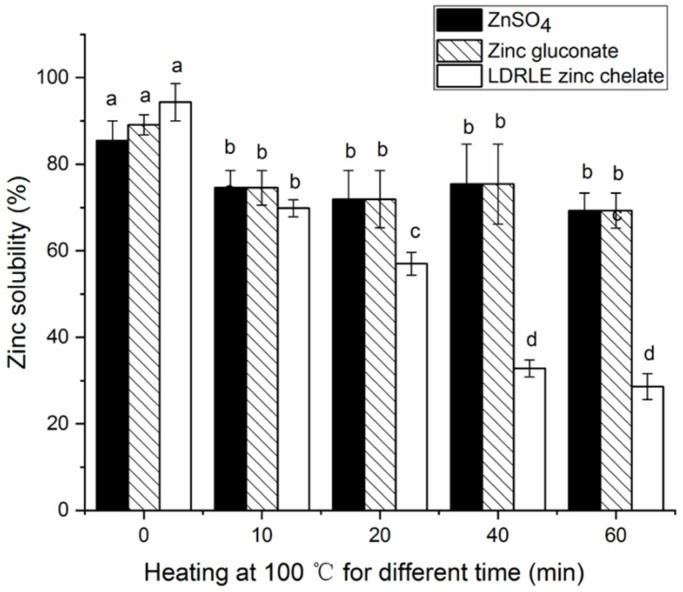
Zinc solubility stability of LDRLE-zinc chelate, zinc sulphate and zinc gluconate against incubation at 100 °C for 10–60 min. Different lowercase letters (**a**–**d**) above the bars or near the lines indicate significant differences (*p* < 0.05).

Zinc solubility is one of the important factors affecting the bioavailability of zinc ions in the body [28]. The zinc-solubility profile of LDRLE-zinc chelate against heating at 100 °C is shown in Figure 7. In general, LDRLE-zinc chelate showed poorer stability in zinc-solubility than zinc sulphate and zinc gluconate. Within the heating time of 10–60 min, the zinc solubility of zinc sulphate and zinc gluconate was not reduced significantly (*p* > 0.05). In contrast, the zinc solubility of LDRLE markedly decreased after incubation at 100 °C for 20–60 min (*p* < 0.05). This result contradicted the results of previous studies [3,6]. Although the chelation with peptides can protect zinc ions against precipitation caused by thermal treatment [3], peptide sequences may be degraded under prolonged heating at high temperatures and the structure of peptide-zinc chelate may collapse, resulting in a reduction in zinc-solubility.

#### 2.5.2. Effect of Various pH Values

**Figure 8:**
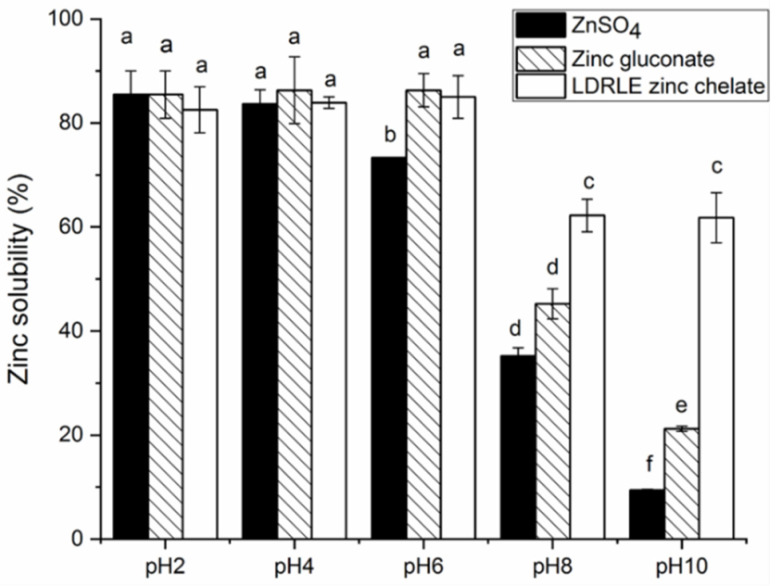
Zinc solubility stability of LDRLE-zinc chelate, zinc sulphate and zinc gluconate under different pH values at 37 °C for 20 min. Different lowercase letters (**a**–**f**) above the bars or near the lines indicate significant differences (*p* < 0.05).

As shown in Figure 8, LDRLE-zinc chelate showed higher zinc solubility under acidic conditions (pH 2.0–6.0) than under alkaline conditions (pH 8.0–10.0) (*p* < 0.05). A similar trend was also observed on zinc sulphate and zinc gluconate. As pH increased, an insoluble zinc slat can form when OH^-^ generated in aqueous solutions reacts with zinc ions [6]. LDRLE-zinc chelate exhibited a higher zinc solubility than zinc sulphate at pH 6.0–10.0 (*p* < 0.05), demonstrating that zinc ions bonding to peptides can be protected effectively from precipitation under alkaline conditions. Moreover, these results reflected that the chelation with LDRLE can improve the stability of zinc ions when they are transported from the stomach (pH 2.0) to the intestine (pH 7.0) [29].

#### 2.5.3. Effect of Gastrointestinal Digestion

**Figure 9:**
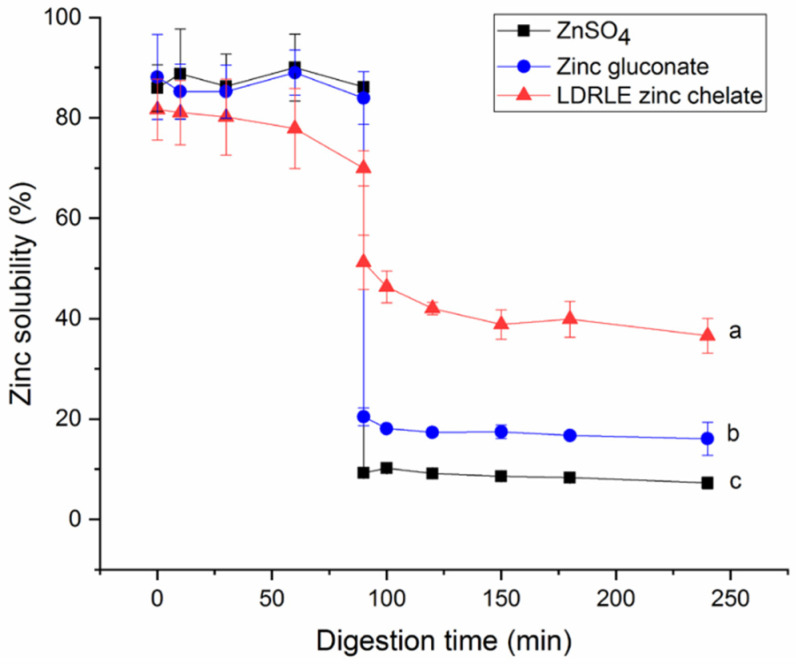
Zinc solubility stability of LDRLE-zinc chelate, zinc sulphate and zinc gluconate against simulated gastrointestinal digestion at 37 °C. The digestion at pH 2.0 during 0 to 90 min, and at pH 2.0 during 91 to 240 min. Different lowercase letters (a–c) near the lines indicate significant differences (*p* < 0.05).

Gastrointestinal digestion is an obstacle that zinc fortifiers need to overcome to exhibit physiological functions in vivo [25]. As shown in Figure 9, zinc solubility decreased significantly after LDRLE-zinc chelate entered the intestinal simulated digestion stage (91–240 min) from the gastric digestion stage (0–90 min) (*p* < 0.05). The same trend was observed on zinc gluconate and zinc sulphate. When zinc ions enter the intestinal tract from the stomach, a part of Zn^2+^ can be converted to insoluble zinc salt with increasing pH value [24]. In the case of LDRLE-zinc chelate, the peptide sequence (LDRLE) may be degraded under gastrointestinal digestion [7], which can weaken the interactions between peptides and zinc ions, leading to a lower zinc solubility. However, further research regarding changes in LDRLE structure under the gastrointestinal digestion is needed. More importantly, LDRLE-zinc chelate showed much higher zinc-solubility than zinc gluconate and zinc sulphate at the intestinal digestion stage (*p* < 0.05), suggesting that the chelation with LDRLE improved stability of zinc ions against gastrointestinal digestion. A similar trend was noted by previous studies [6,26].

### 2.6. Zinc Transportation across Caco-2 Cells

**Figure 10:**
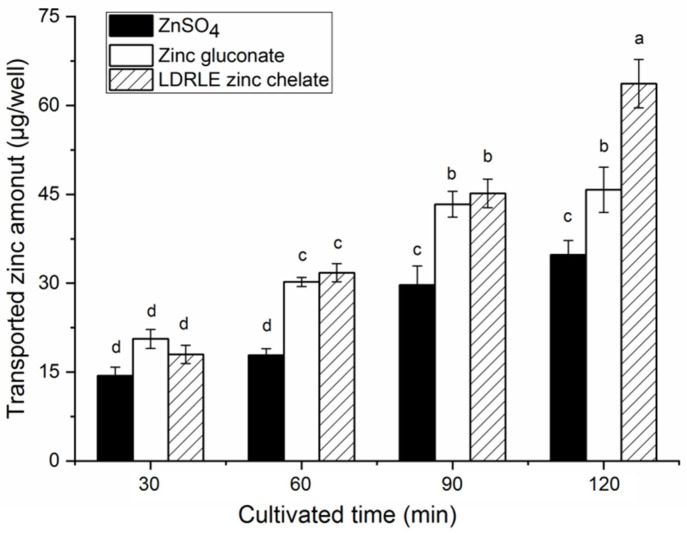
Zinc transport contents at the basolateral sides of Caco-2 cell monolayers of LDRLE-zinc chelate, zinc sulphate and zinc gluconate. Different lowercase letters (**a**–**d**) above the bars indicate significant differences (*p* < 0.05).

The result of the current study demonstrated that ZnSO_4_, zinc gluconate and LDRLE-zinc chelate had no significant (*p* > 0.05) cytotoxicity toward Caco-2 cells. As shown in Figure 10, LDRLE-zinc chelate showed a higher zinc transport amount than zinc sulphate from an incubation time of 60 min (*p* < 0.05); while the chelate showed a higher transport amount than zinc gluconate at 120 min (*p* < 0.05), suggesting that LDRLE-zinc chelate can improve zinc transportation across the intestinal membrane [27]. One of the reasons was that, compared to zinc sulphate and zinc gluconate, LDRLE-zinc chelate had a higher zinc solubility than ZnSO_4_ and zinc gluconate under pH 6.0–8.0 (Figure 8) and gastrointestinal digestion (Figure 9). When zinc fortifiers move from the stomach to the intestine, the increased pH value and gastrointestinal digestion both reduce the zinc solubility of the zinc fortifiers, resulting in poor zinc transportation [28]. Chelation with PDRLE improved solubility of zinc ions under pH 6.0–80 and gastrointestinal digestion, so LERLE showed a higher ability for zinc transport. However, there is much work to be done to investigate the absorption and transportation mechanism of the chelate in vivo.

## 3. Materials and Methods

### 3.1. Materials

Sweet almond (*Prunus armeniaca* L.) expeller was donated by XingBao Farmer Cooperation, Qixian, China. Caco-2 cells were purchased from the Chinese Academy of Sciences (Shanghai, China). Dulbecco’s modified Eagle’s medium (DMEM) and Hank’s balanced salt solution (HBSS) buffer were purchased from SJQI Biotech. Co., Ltd. (Wuhan, China). Flavourzyme (from *Aspergillus oryzae*, 2 × 10^4^ U/g), trypsin (from bovine pancreas, 5 × 10^4^ U/g), pepsin (from porcine stomach, 5 × 10^4^ U/g) and Alcalase (from *Bacillus licheniformis*, 2 × 10^5^ U/g) were purchased from Huke Co. (Tianjin, China). Zinc sulphate, 4-(2-Pyridinazo)-resorcinol (PAR), 3-(4,5-dimethylthiazol-2-yl)-2,5-diphenyltetrazolium bromide (MTT), Dithiothreitol (DTT) and other analytical grade chemicals were bought from Dikun Chemical Co. (Qingdao, China).

### 3.2. Preparation of Sweet Almond Cake Amandin Hydrolysates

Following the modified method of Souza et al. [30], sweet almond expeller was ground and passed through a 40-mesh sieve. The powder was degreased using *n*-hexane (1:15, *m*/*v*) two times. The obtained defatted powder (25 g) was dispersed into 20 mmol/L of Tris-HCl buffer (pH 8.0, 500 mL) and then stirred at 35 °C and 175 r/min for 120 min. Afterwards, the dispersions were filtered with filter paper and the filtrate solution was pooled and centrifuged at 9500× *g* for 25 min. The supernatant was pooled and adjusted to pH 3.5 with 0.1 mol/L of HCl or 0.1 mol/L of NaOH, and then incubated at 4 °C overnight. After centrifugation at 6000× *g* at 4 °C for 35 min, the precipitate was collected and dialyzed against deionized water at 4 °C for 8 h. Then, the dialysate was lyophilized and sweet almond expeller amandin (SAEA) was obtained.

Sweet almond expeller amandin hydrolysates (SAEAH) were prepared using a two-stage Alcalase-Flavourzyme hydrolysis process following the description of Liu et al. (2016). The obtained SACA (2 g) was dispersed in 20 mmol/L of Tris-HCl buffer (90 mL) and adjusted to pH 8.0 with 0.1 mol/L NaOH, and then Flavourzyme (0.05 g) and Alcalase (0.1 g) were added. The mixture was stirred in a water bath (120 r/min) at 50 °C for 125 min, and heated in boiling water for 8 min to deactivate the enzymes. Afterwards, the reaction solution was centrifuged at 15,500× *g* and 4 °C for 16 min using the TDL-20 centrifuge. The supernatant was pooled and freeze-dried to obtain sweet almond expeller amandin hydrolysates (SAEAH). In addition, the trinitrobenzenesulfonic acid method was employed to determine the degree of hydrolysis [31].

### 3.3. Purification of SAEAH

SAEAH was resolved in ultrapure water (2 mg/mL) and passed through a W-45 ultrafiltration membrane (0.45 μm, Daning Co., Dalian, China). The filtrate was purified using gel column chromatography (Φ1.2 × 80 cm) with Sephadex G-15 as the stationary phase and distilled water as elution solution (2.6 mL/min). The monitored wavelength was 220 nm. After a five-minute interval, the effluent fractions were collected, lyophilized (with a LGJ-10N freeze-dryer, Keya Instrument Co., Beijing, China) and subjected to determination of their zinc-chelating capacity. Subfractions of excellent zinc-chelating ability were isolated using reversed-phase high-performance liquid chromatography (RP-HPLC). The RP-HPLC isolation was conducted with a Zorbax analytical C_18_ column (4.6 × 250 mm, Agilent Technologies, Palo Alto, CA, USA). The elution solvent contains acetonitrile (mobile phase B) and trifluoroacetate (mobile phase A, 0.1%, *v*/*v*). From 0 to 30 min, the RP-HPLC isolation was performed with increasing concentrations of acetonitrile (from 5% to 35%, *v*/*v*), and then performed with a constant concentration of acetonitrile (35%, *v*/*v*) for 10 min. The monitored wavelength was 220 nm. Subfractions corresponding to the elution peaks were separately pooled and lyophilized, and then used for analysis of their zinc-chelating ability. Peptide sequences of the subfractions possessing excellent zinc-chelating ability were analysed.

### 3.4. Zinc-Chelating Ability Assay

Zinc-chelating ability was determined using the PAR colorimetric method [32]. Briefly, 250 μL sample solution (dissolved in 0.1 mol/L of HEPES-KOH buffer) were mixed with 125 μL DTT (8 mmol/L), 125 μL zinc sulphate (ZnSO_4_, 250 μmol/L) and 2 mL ultrapure water. The mixed solution was stirred (180 r/min) at 37 °C for 10 min, and then 250 μL PAR (0.2 mmol/L, pH 7.5) was added. After incubation at 37 °C for 3 min, the absorbance at 500 nm was read. Zinc content was quantified by regression of the zinc sulphate standard curve (A = 0.0901n(C) + 0.1012; R^2^ = 0.9802; where A represents the absorbance at 500 nm, and C is the concentration of Zn^2+^, μg/ mL). Zinc-chelating capacity was calculated as follows:

(1) $$Zinc\;chelating\;ability\;\left(mg/g\right)=\left(C_{c}-C_{s}\right)\times \;65.38\times 10^{-3}\times V\;/M$$

where, *C_c_* (μmol/L) is the zinc concentration in the reaction solution without samples; *C_s_* is the zinc concentration in reaction solution after the chelating reaction (μmol/L); *V* is the volume of the reaction solution; *M* is the mass of the samples (g); and 65.38 is the mol mass of zinc (g/mol).

### 3.5. Identification, Synthesize and Physicochemical Characteristics of Peptide Sequence

Identification of the SAEAH peptide sequence was conducted on a hybrid quadrupole orbitrap mass spectrometer (Q Exactive, Thermo Fisher, Bremen, Germany) using the same parameters as described by Xu et al. [33]. The obtained mass spectrometry data were analysed using Peak-Studio-7.5-De-Novo™ software (Bioinformatics Solutions, Inc., Waterloo, Canada). Moreover, the obtained amino acid sequences were verified using the National Center for Biotechnology Information database (Bethesda, MD, USA). Chemical synthesis of peptide sequences was done in Yaoshan Biological Tech. Co. (Shaoxing, China). In addition, physicochemical characterization of the obtained peptide sequences was conducted using the database AHTPDB (http://crdd.osdd.net/raghava/ahtpdb/, accessed on 21 April 2022) [34].

### 3.6. Toxicity Evaluation

SAEAH peptides’ toxicity was evaluated using the database ToxinPred (http://www.imtech.res.in/raghava/toxinpred/, accessed on 5 May 2022) according to the description of Sudheer et al. [35]. 

### 3.7. Preparation of Peptide-Zinc Chelate

Preparation of peptide-zinc chelate was conducted following the description of Sun et al. [6]. Briefly, SAEAH peptide was mixed with 250 μmol/L ZnSO_4_·7H_2_O (25:1, *m*/*m*) and adjusted to pH 7.6. The reaction solution was stirred (135 r/min) at 27 °C for 50 min, and then centrifuged at 4500× *g* for 25 min. The supernatant was pooled and precipitated with anhydrous ethanol (1: 4, *v*/*v*) at 4 °C for 30 min. After centrifugation at 12,000 × *g* for 12 min, the precipitate was lyophilized and SAEAH peptide-zinc chelate was obtained. 

### 3.8. Structural Characteristics

#### 3.8.1. Scanning Analysis with Ultraviolet Wavelength

SAEAH peptide-zinc chelate (1 mg/mL, dissolved in ultrapure water) was scanned with an ultraviolet wavelength scanner (UV759CRT, Chuangju Instrument Co., Qingdao, China). The range of scanned wavelength ranged from 190 to 440 nm [36]. Pure SAEAH peptide (1 mg/mL) and zinc sulphate (1 mg/mL) were used as comparisons.

#### 3.8.2. Fourier-Transform Infrared Spectroscopy (FT-IR)

Briefly, dry KBr (around 0.1 g) was blended thoroughly with SAEAH peptide-zinc chelate or purified SAEAH peptide (2 mg). The mix powder was ground and pelleted into a table of 1–2 mm, and then scanned using a FT-IR-850 spectrometer (Atomic Instruments, Suzhou, China). The scanning wavenumbers ranged from 4000 to 400 cm^–1^.

#### 3.8.3. Surface Microstructure Analysis

After being sprayed with a 10 nm-thick layer of gold, the microstructure of SAEAH peptide-zinc chelate was investigated with a 7500F scanning electron microscope (JSM, Tokyo, Japan) [33]. Micrographs were taken with a scale bar of 1 μm. Moreover, the magnification was 5000× and the acceleration voltage was 10 kV, respectively.

### 3.9. Stabilities

#### 3.9.1. Thermal Stability

SAEAH peptide-zinc chelate solution (1 mg/mL, dispersed in ultrapure water) was incubated in boiling water for 10, 20, 40 and 60 min [37]. After centrifugation at 6000× *g* for 15 min, the supernatant was collected for zinc content determination. The untreated chelate was used as a control, while zinc sulphate (100 μg/mL) and zinc gluconate (100 μg/mL) were used as comparisons. Zinc solubility was calculated as follows:

(2) $$Zinc\;solubility\;\left(\%\right)=Zinc\;in\;supernatant/Total\;zinc\;in\;solution\;\times 100\;\;$$

#### 3.9.2. Zinc Solubility at Different pH Values

Effects of different pH values (2.0–10.0) on zinc-solubility of SAEAH-zinc chelate were investigated using the same procedure as Xu et al. [33]. Zinc content of the chelate solution before and after each treatment was determined using the PAR method [24], and zinc solubility was calculated by Equation (2). Both zinc sulphate (100 μg/mL) and zinc gluconate (100 μg/mL) were used as comparisons.

#### 3.9.3. Effect of the Gastrointestinal Digestion

Zn solubility of SAEAH-zinc chelate against simulated gastrointestinal digestion was investigated according to the description of Xu et al. [33]. Briefly, SAEAH peptide-zinc chelate was first treated with the simulated gastric fluid (composed of 4.5 mg/mL of Trypsin, 62.5 mg/mL of NaHCO_3_ and 30 mg/mL of pig bile salt) at 37 °C and pH 2.0 for 90 min. Then the chelate was treated with the simulated intestinal fluid containing pepsin (0.4 mg/mL) and NaCl (8.77 mg/mL) at 37 °C and pH 6.8 for 150 min. During the simulated gastrointestinal digestion, 1 mL of the reaction solution was sucked out at 0, 10, 30, 60, 90, 120, 150, 180 and 240 min, respectively, and then incubated in boiling water for 6 min. After incubation at 4500× *g* for 25 min, the supernatant was pooled and the zinc content was determined. Based on this, zinc solubility was calculated using Equation (2). Both zinc sulphate (100 μg/mL) and zinc gluconate (100 μg/mL) were used as comparisons.

### 3.10. Zinc Transport across Caco-2 Cells

As per the method of Wang et al. [2] with some modifications, Caco-2 cells were seeded on Transwell plates (1.5 × 10^5^ cells/cm^2^) and cultured in DMEM containing penicillin–streptomycin–neomycin mixture (1 mg/mL) and foetal bovine serum (20 mg/mL) at 37 °C in a humidified atmosphere of 5% CO_2_. The transepithelial electrical resistance (TEER) was determined using Millicell-ERS-00002 system (Millipore Co., Burlington, MA, USA). A cell monolayer was established if TEER was more than 400 Ω·cm^2^. Then 0.6 mL of HBSS buffer (without calcium and magnesium) was added to both the apical (AP) side and the basolateral (BL) side. After incubation at 37 °C for 30 min, the HBSS buffer was sucked out. Immediately, peptide-zinc chelate (300 μg/mL, dissolved in HBSS buffer) was added to the AP side (0.4 mL/well), whereas HBSS buffer was added to the BL side (0.6 mL/well). The cells were cultured at 37 °C for 120 min. At 30 min intervals, 50 μL of sample solution was sucked out from the BP side for zinc content determination [32], and 50 μL of HBSS was filled up immediately. Zinc transported was calculated as the amount of zinc at the BP side [2]. Zinc content was determined using the PAR colorimetric method [24]. Zinc gluconate and zinc sulphate (5 mmol/ L) were used as comparisons, while the blank control was only treated with HBSS buffer. In addition, effects of ZnSO_4_/zinc gluconate and peptide-zinc chelate on cell viability were measured using the MTT method [38].

### 3.11. Data Analysis

All the tests were carried out in triplicate at least (*n* ≥ 3). The significance of differences among data was analysed with a one-way analysis of variance. Multiple comparisons were carried out using IBM SPSS Statistics software (Version 16, Chicago, IL, USA) with a significant level at *p* < 0.05.

## 4. Conclusions

Two novel peptides VDLVAEVPRGL and LDRLE were identified in sweet almond expeller amandin hydrolysates. LDRLE of excellent zinc-chelating capacity (24.73 mg/g) was selected to prepare peptide-zinc chelate. Both the amino group and carboxyl group of LDRLE were the main bonding sites for zinc ion. Moreover, the chelation with LDRLE improved significantly zinc-solubility of zinc sulphate under pH 6.0–10.0 and gastrointestinal digestion (*p* < 0.05). In addition, LDRLE-zinc chelate showed a higher capacity to improve zinc transportation than zinc sulphate and zinc gluconate (*p* < 0.05). These results shed light on applications of peptides identified in sweet almond expeller amandin hydrolysates as ingredients of functional foods to improve zinc bioavailability.

## Figures and Tables

**Table 1:** Amino acid sequences, antioxidant activity, physicochemical characteristics and toxicity of peptides identified in sweet almond cake amandin hydrolysates.

Peptide Sequence	LDRLE	VDLVAEVPRGL	LDRLE-Zinc Chelate	EDTA ^a^
Molecular weight (Da)	644.77	1164.45		ND
Matched sequence in *Prunus armeniaca* ^b^	T.LDRLE.V	T.VDLVAEVPRGL.G		ND
Zinc chelating capacity (mg/g)	24.73 ± 2.45 ^f^	4.33 ± 0.33 ^e^		57.17 ± 2.63 ^d^
Hydrophilic amino acid content (%)	60.00	36.36	60.00	ND
Hydrophobicity ^c^	−0.41	−0.08		ND
Amphiphilicity	0.74	0.75		ND
Hydrophilicity	1.08	0.40		ND
Isoelectric point	4.38	4.26		ND
Toxicity	Non-Toxin	Non-Toxin	Non-Toxin	ND

^a^ EDTA and glutathione were used as positive control for zinc chelating capacity and antioxidant activity, respectively. ^b^ From the National Center for Biotechnology Information (NCBI). ^c^ Physicochemical characteristics and potential toxicity were predicted separately using the AHTPDB database (http://crdd.osdd.net/raghava/ahtpdb/, accessed on 21 April 2022) and the database ToxinPred (www.imtech.res.in/raghava/toxinpred/, accessed on 5 May 2022). ND: not measured. Different lowercase letters (^d–f^) in the same line denote significant difference (*p* < 0.05).

## Data Availability

Not applicable.

## Sample Availability

Samples of the compounds are not available from the authors.
